# Facilitating relaxation and stress reduction in healthy participants through a virtual reality intervention: study protocol for a non-inferiority randomized controlled trial

**DOI:** 10.1186/s13063-022-06307-8

**Published:** 2022-05-09

**Authors:** Miriam Kampa, Johannes Finke, Tobias Stalder, Leandra Bucher, Holger Klapperich, Fabian Mertl, Christian Zimmer, Christian Geiger, Marc Hassenzahl, Tim Klucken

**Affiliations:** 1 Department of Clinical Psychology and Psychotherapy, University of Siegen, Obergraben 23, 57072 Siegen, Germany; 2 Bender Institute of Neuroimaging, Justus Liebig University, Giessen, Otto-Behaghel-Str. 10 H, 35394 Giessen, Germany; 3 Ubiquitous Design/Experience and Interaction, University of Siegen, Kohlbettstraße 15, 57072 Siegen, Germany; 4 Mixed Reality and Visualization (MIREVI), Faculty of Media, University of Applied Sciences Düsseldorf [M1], Münsterstraße 156, 40476 Düsseldorf, Germany

**Keywords:** Virtual reality, Relaxation, Stress reduction, Stress test, Randomized controlled trial

## Abstract

**Background:**

Repeated or chronic stress is considered a major source of disease, in terms of both somatic and mental illnesses. The prevention of stress-related disease by interventions for relaxation has thus increased societal relevance. In this randomized controlled non-inferiority trial, we will compare a newly developed virtual reality (VR) environment for relaxation to an active control group applying a freely chosen relaxation method. To test if our VR environment supports relaxation in a situation of acute stress, a standardized stress induction protocol will precede the relaxation phase.

**Methods:**

One hundred healthy participants will be recruited from the University of Siegen and randomly assigned to the VR or the active control group that will be free to choose their own relaxation strategy. The multi-sensory VR includes visual, acoustic, and haptic features to induce a strong feeling of presence. The laboratory testing will comprise a baseline measurement, a stress induction, a relaxation intervention, and a recovery measurement. The primary outcomes are self-reported stress and relaxation measured with a visual analog scale (VAS) at pre- and post-baseline, at the start, middle, and end of the stress induction, at pre- and post-relaxation, at pre- and post-recovery, and in the evening of testing. Secondary outcomes are the physiological parameters, namely heart rate and heart rate variability, tonic skin conductance level as well as the number of non-specific skin conductance responses, systolic and diastolic blood pressure and respiratory rate recorded during the four experimental phases as well as state mood, and state rumination assessed at four time points (pre- and post-stress, post-relaxation, and in the evening of testing). Finally, post-event processing will be assessed after relaxation and in the evening of testing. Repeated measures ANOVAs will be performed to test for statistical effects of group, time, and group × time interaction.

**Discussion:**

The newly developed, multi-sensory VR offers an intervention for relaxation without prior training. Its immersive character might increase efficacy compared to other relaxation methods, especially in situations of acute stress. Future directions could be the development of a mobile version of the VR to enhance accessibility for users. To achieve a transfer of training effects to real life, VR components should successively be eliminated until relaxation is practiced without guidance by the VR.

**Trial registration:**

ISRCTN Registry ISRCTN11162338. Retrospectively registered on January 22, 2021

## Administrative information

Note: The numbers in curly brackets in this protocol refer to SPIRIT checklist item numbers. The order of the items has been modified to group similar items (see http://www.equator-network.org/reporting-guidelines/spirit-2013-statement-defining-standard-protocol-items-for-clinical-trials/).

**Table:** 

Title {1}	Facilitating relaxation and stress reduction in healthy participants through a virtual reality intervention: study protocol for a non-inferiority randomized controlled trial
Trial registration {2a and 2b}.	Retrospectively registered with ISRCTN Registry (Trial ID: ISRCTN11162338, https://www.isrctn.com/ISRCTN11162338) on January 22, 2021.
Protocol version {3}	Issue date: 5th April, 2022, Protocol Version: 2.0 Revision chronology: - Original version V1.0: 15th March, 2021 Amendment 01: 5th April, 2022. We restructured our manuscript according to the *Trials* structured study protocol template and included missing information on SPIRIT items as requested by the reviewers. We adjusted the power analysis for the non-inferiority design.
Funding {4}	This work is funded by the Bundesministerium für Bildung und Forschung (BMBF, Funding number: 16SV8068).
Author details {5a}	Miriam Kampa^1, 2, *,^ Johannes Finke^1^, Tobias Stalder^1^, Leandra Bucher^1^, Holger Klapperich^3^, Fabian Mertl^4^, Christian Zimmer^4^, Christian Geiger^4^, Marc Hassenzahl^3^, Tim Klucken^1^ 1Department of Clinical Psychology and Psychotherapy, University of Siegen, Obergraben 23, 57072 Siegen, Germany;2Bender Institute of Neuroimaging, Justus Liebig University, Giessen, Otto-Behaghel-Str.10H, 35394 Giessen, Germany;3Ubiquitous Design/Experience and Interaction, University of Siegen, Kohlbettstraße 15, 57072 Siegen, Germany;4Mixed Reality and Visualization (MIREVI), Faculty of Media, University of Applied Sciences Düsseldorf [M1], Münsterstraße 156, 40476 Düsseldorf, Germany; *Corresponding author: Miriam Kampa (miriam.kampa@uni-siegen.de) MK and TK designed the study and wrote the manuscript. MK prepared all the materials and instructions for the data acquisition. JF and TS supported the technical side of the data acquisition. LB programmed the online questionnaires. HK developed the narrative and the design of the rationale for the VR. FM and CNZ programmed the VR. CG and MH are supervisors who contributed to the development of the VR. MH obtained the grant. All authors have made contributions to the design of the study and writing of the manuscript and have read and approved the final version of the manuscript. No professional writers have been involved.
Name and contact information for the trial sponsor {5b}	Bundesministerium für Bildung und Forschung (BMBF), VDI/VDE Innovation + Technik GmbH, Alexander ReissSteinplatz 1, 10623 Berlin, Germany, Tel.: +49 30 310078-268, alexander.reiss@vdivde-it.de, www.vdivde-it.de.
Role of sponsor {5c}	The funding source was not involved in the study design; the collection, analysis, and interpretation of the data; the writing of the report; or the decision to submit the article for publication.

## **Introduction**

### Background and rationale {6a}

Repeated or chronic stress is considered a major source of disease, in terms of both somatic and mental illnesses [1]. Stress-related mental disorders do not only lead to subjective suffering but are associated with large economic costs due to health care expenditures, absenteeism, and inability to work [2, 3]. The prevention of stress-related diseases by methods of stress reduction and relaxation techniques has thus an increasing societal relevance. Common relaxation practices, like mindfulness-based stress reduction and meditation techniques, achieve small to moderate efficacy rates [4–6]. They require repeated practice and the capacity to retrieve and apply them in situations of high distress.

Emerging computational power and advances in techniques have rendered the use of virtual realities (VRs) accessible and VRs increasingly immersive [7]. A VR is a 3D simulation of a natural environment that participants can explore by looking or even moving around. New VR systems with a high resolution, a broad field of view, head tracking, and stereo sounds achieve a high level of immersion, i.e., they enable natural sensorimotor perceptions in the VR. They thus have the potential to induce a strong feeling of presence and to shield participants’ attention from distraction [8]. VRs are increasingly used in the field of clinical psychology and behavioral medicine [9, 10]. Applications range from exposure therapy in patients with anxiety disorders, to addiction-related interventions to practices for mindfulness [8–12]. Within the VR, participants can find themselves in a safe space wherein they can practice a specific behavior. An advantage is that a multi-sensory VR for stress reduction does not require prior training but directly targets the process level and could train participants’ relaxation ability online.

A VR can target relaxation processes by different mechanisms. In biofeedback, training participants receive online feedback on their physiological responses (e.g., heart rate variability) to learn how to regulate those bodily stress responses [13–16]. Another way VRs can target relaxation processes is by supporting the practice of mindfulness [8, 12, 17]. VRs of this type often use nature scenes combined with nature sounds as a setting [8, 12, 16, 18, 19]. In addition, the feeling of presence generated by the VR could facilitate practicing state awareness, a basic element of mindfulness [8, 12]. In addition, VRs can also serve emotion regulation by distraction which can reduce symptoms of anxiety, depression, fatigue, and pain [20].

Significant effects of VR relaxation interventions have predominantly been found in the domain of self-reported emotional states and less so in physiological measures [16, 19, 21]. VR relaxation interventions led to reduced subjective stress [16], anxiety, and depression [15, 20, 21], as well as reduced negative affect [8, 18, 19], increased state relaxation [8, 21] and relaxation self-efficacy [13], increased recreation [19], and enhanced positive affect [12, 19].

In the current randomized controlled non-inferiority trial, we will test a newly developed VR for relaxation against an active control group applying a freely chosen relaxation method. We hypothesize non-inferiority of the VR, assuming that the VR is equally effective as a freely chosen, preferred, and well-practiced relaxation method. So far, the majority of studies evaluated the respective relaxation intervention in a calm and comforting setting. This raises the question, if participants will be capable to use the respective relaxation intervention also in stressful situations, when the intervention would be most needed and processing resources are limited. We therefore implement the VR not only for relaxation but to downregulate stress induced before the relaxation phase.

### Objectives {7}

Our main objective is to investigate if the VR intervention is non-inferior to an active control group, applying a freely chosen relaxation method, in inducing stress reduction and relaxation indicated by ratings and physiological measures.

### Trial design {8}

In the current non-inferiority trial, a randomized controlled design is applied, wherein participants will be randomly assigned to one of two parallel groups: either the virtual reality intervention (VR) or an active control group. The allocation ratio will be 1:1. To test the effect of stress reduction, stress will be induced before the relaxation phase (see the description of the procedure for more details).

## Methods: participants, interventions, and outcomes

### Study setting {9}

Data collection will take place at the University of Siegen (Germany) in the laboratories of the Department of Clinical Psychology and Psychotherapy.

### Eligibility criteria {10}

We will recruit participants aged 18 to 30 years. The eligibility criteria will be no current psychiatric or cardiovascular disease and no current intake of psychoactive or neuroactive medication. Furthermore, participants should not have experienced a trauma that could possibly be reactivated by the negative images that will be shown in the stress task

### Who will take informed consent? {26a}

Informed written consent will be obtained by the responsible experimenter of the trial management group.

### Additional consent provisions for collection and use of participant data and biological specimens {26b}

Participant data will not be used in ancillary studies. This trial does not involve collecting biological specimens for storage.

### Interventions

#### Explanation for the choice of comparators {6b}

As a comparator, we want to use a strategy for relaxation that is subjectively perceived as most effective. The active control group will be free to choose their own method for relaxation that can be conducted in a sitting position and involves low levels of physical activity. The comparator is not associated with any risks for the participants.

#### Intervention description {11a}

##### Procedure

**Fig. 1:**
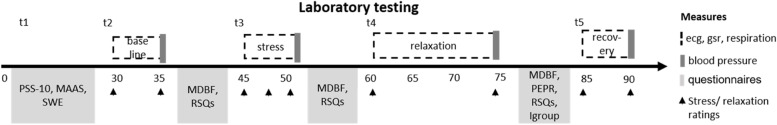
Procedure for laboratory testing session. Time points t1 to t5 from the schedule of enrollment are given as orientation. Time point t6 will be collected in the evening of the laboratory testing and is not included here. ECG, electrocardiogram; GSR, galvanic skin conductance response; Igroup, questionnaire on experienced presence in the VR; MAAS, Mindful Attention and Awareness Scale; MDBF, Mood State Questionnaire; PEPR, post-event processing; PSS-10, Perceived Stress Scale; RSQs, Response Style Questionnaire – state rumination; SWE, Self-Efficacy Questionnaire

Study participation will consist of an online pre-intervention and an online post-intervention survey taking approximately 2 h in total and one laboratory testing of approximately 1.5 h. Online surveys were set up in the online survey tool lime survey (LimeSurvey GmbH, Hamburg, Germany). The pre-intervention survey will assess chronic stress (TICS, [22]), depression, anxiety, and stress (DASS, [23]) as well as trait rumination (RSQ, [24–26]). Testing sessions will take place in the laboratories of the Department of Clinical Psychology and Psychotherapy at the University of Siegen. At the beginning of the testing session, participants will give their informed written consent and will fill out questionnaires on a computer for approximately 30 min. These questionnaires assess perceived stress (PSS-10, [27]), self-efficacy (SWE, [28]), and mindful attention and awareness (MAAS, [29]). The testing session will comprise an initial 5-min baseline measurement, a 5.5-min stress induction by the Mannheim Multicomponent Stress Test (MMST, [30]), subsequently a 15-min relaxation phase, and finally a 5-min recovery measurement. During all of these phases, physiological data will be recorded, namely an electrocardiogram (ECG), galvanic skin conductance response (GSR), respiration, and blood pressure (see description on data collection and management, SPIRIT item 18a for more details). Stress and relaxation ratings will be measured with a visual analog scale (VAS) on a Likert scale ranging from 0 (not stressed, not relaxed) to 9 (very stressed, very relaxed) at pre- and post-baseline; at the start, middle, and end of the stress induction; at pre- and post-relaxation; at pre- and post-recovery; and in the evening of testing. Additionally, two questionnaires on state mood (MDBF, [31, 32]) and state rumination (RSQs, adaptation of RSQ for state [24–26]) will be repeatedly administered at post-baseline, post-stress, and post-relaxation as well as in the evening of testing. Post-event processing (PEPR, translated to German, [33]) will be assessed after relaxation and in the evening of testing. Experienced sense of presence in the VR will be assessed directly after relaxation [34]. Figure 1 shows the procedure for the laboratory testing session.

##### Virtual reality setup

**Fig. 2:**
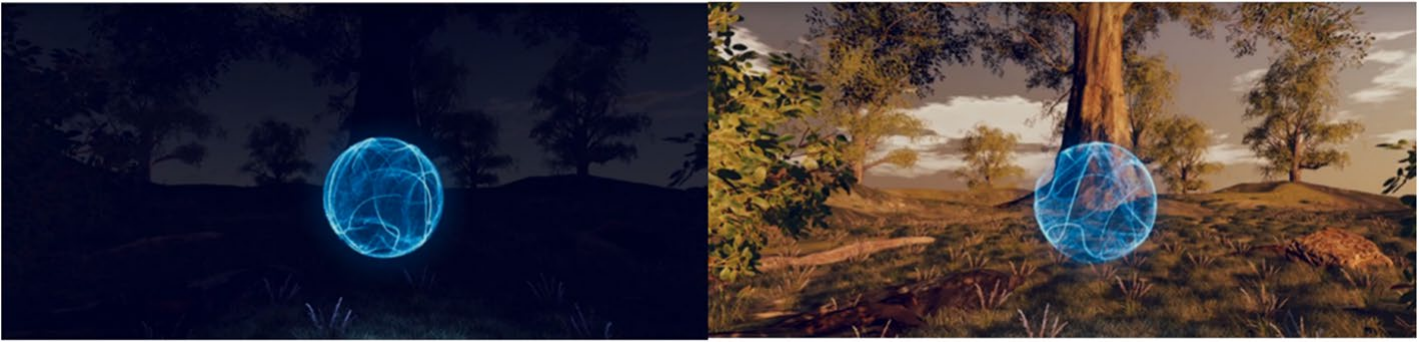
Illustration of the VR environment. Beginning during night and ending during daylight. The blue sphere changes in size for the intervention on respiration increasing in size for inhalation and shrinking for exhalation

The multi-sensory VR was developed in cooperation with the Faculty of Media at the University of Applied Sciences in Düsseldorf, the chair of Ubiquitous Design/Experience and Interaction at the University of Siegen, the Department of Clinical Psychology of the University of Siegen, and industrial cooperation partners (LAVAlabs Moving Images GmbH & Co. KG, Düsseldorf; Medisana Space Technologies GmbH, Düsseldorf; tro GmbH, Düsseldorf; IOX GmbH, Düsseldorf). The VR includes visual, acoustic, and haptic features and is accompanied by a relaxing narrative. The VR environment comprises a landscape with fields and trees. At the beginning of the scene, the landscape is shown during the night. Participants will be able to see stars in the sky if they turn their heads upwards. During the course of the 14-min relaxation, sunrise is simulated with colors of the landscape changing from dark via orange sunrise to daylight. In front of the landscape an airy, blue sphere depicts a pattern of respiration by increasing for inhalation and shrinking for exhalation. See Fig. 2 for an illustration of the VR environment.

The VR will be presented using the HTC Vive pro HMD with a resolution of 2880 × 1600 pixels and a refresh rate of 90 Hz (HTC Corporation, ViveTM). The HTC Vive infrared sensor will be used to transfer participants’ head movements to the VR, thereby enhancing immersion. The VR is implemented using the Unity3D engine, which applies the C# language. A Nexus-10 MKII device (Mind Media, Herten, The Netherlands) will be used to continuously monitor the participant’s respiratory rate via a respiration belt tied around the chest.

During the first part of the VR intervention, the participant will have the possibility to familiarize with the VR environment, to listen to the introduction by the narrator, and to observe one’s own respiration. The sphere will not change in size and thus not reflect any respiratory pattern. During the second part of relaxation, starting with the fourth minute of the VR intervention, the blue sphere will increase in size for inhalation and shrink for exhalation. The respiratory rate simulated by the blue sphere will be adapted to the participant’s respiratory rate and slowed down to guide the participant’s respiration. This will be accomplished using a mapping algorithm. To calculate the decelerated respiratory rate for each participant, the duration of the participant’s respiratory cycles will be assessed during the first part and divided by two to approximate the duration of inhalation and exhalation. The duration of inhalation and exhalation will then each be mapped from values between 0 and 9 s to values between 4 and 9.5 s. This results in higher initial respiratory rates being more affected by the deceleration than lower initial respiratory rates. To achieve a longer exhalation than inhalation, 3 s is added to the computed exhalation duration. During the third part of the VR intervention, after 6 min, the user will be requested to hold the breath for 2 s following each inhalation and to exhale long and deeply.

Via headphones, participants will listen to calming music and nature sounds. In addition, a narrator will give instructions on relaxation and respiration. During the relaxation phase, participants will be seated on a cantilever chair with a massage mat (Medisana Space Technologies GmbH, Düsseldorf) placed on its surface. The massage mat will be automatically triggered by the Unity3D engine at the start of the VR. Pressure will be circularly applied to the back for a massage. In addition, heating elements will spread warmth to the back during massage. At the completion of the VR, the mat will strike down the back. As an additional feature, participants will be asked to place a custom-made pillow in their lap that responds synchronously to the increasing size of the blue sphere representing inhalation. Vibrations will start at inhalation onset, increase to a maximum and then decrease until the peak of inhalation. No vibrations will be given during the shrinking sphere representing exhalation.

##### Active control group

In the active control group, participants will be free to choose a method for relaxation that is subjectively perceived as effective. Participants will be instructed to decide on a strategy and to keep it during the 15-min relaxation phase. Some suggestions will be given to the participants for orientation; these are listening to music, reading, applying breathing exercises, or meditation. Participants will be encouraged to use their own relaxation method in the instructions. The only posed restrictions will be that it is unrelated to physical activity and can be performed in a sitting position. The applied strategies will be assessed via self-report after the relaxation phase.

##### Mannheim Multi Component Stress Test (MMST)

The MMST applies stressors of different modalities (cognitive, acoustic, emotional, motivational) and has been shown to reliably induce a physiological and subjective stress response [30, 35]. The MMST was programmed in the software Presentation (NBS, San Francisco, USA). As a cognitive stressor, mental arithmetics have to be performed under time pressure (PASAT-C [36];) consisting of the addition of two sequentially presented numbers in the range of 0 to 20. Numbers are shown for 250 ms each with a time latency between numbers of 3 s in the first and 2 s in the second half of the task. Answers have to be marked with the mouse on a numerical keypad presented in the lower part of the screen. Participants will continuously receive performance feedback on the total number of correct answers. As an emotional stressor, 44 negative, fear- and disgust-related pictures are presented for 5 s each as background during the PASAT-C. Five negative pictures are always followed by one positive picture for 3 s to avoid habituation. To direct participants’ attention to the pictures, they have to detect pictures presented twice during an initial 1-min phase before the start of the actual PASAT-C. White noise and 60 randomly timed explosion sounds presented over headphones serve as an additional acoustic stressor. During the first minute of the task, the noise volume is kept constant (75 dB), while with the onset of the PASAT-C, the volume starts to continuously rise to a maximum of 93 dB. As an additional acoustic stressor, explosion sounds are given for incorrect answers. Finally, to induce motivational stress, participants will be told that their reimbursement will be reduced if they perform too poorly.

### Criteria for discontinuing or modifying allocated interventions {11b}

There will be no special criteria for discontinuing or modifying the allocated interventions.

### Strategies to improve adherence to interventions {11c}

Face-to-face instructions will be given throughout the laboratory testing session to increase adherence to the intervention protocol. Adherence to the relaxation instructions will be assessed immediately after the intervention with a short questionnaire. Self-reports and physiological data will be acquired throughout the testing session to monitor the participants’ responses to the study protocol.

### Relevant concomitant care permitted or prohibited during the trial {11d}

Trial participation will not require alteration to usual care pathways (including the use of any medication) in either of both trial arms.

#### Provisions for post-trial care {30}

There is no anticipated harm, and there are no provisions for post-trial care. After study participation, participants will receive compensation in the form of 15 Euros monetary reimbursement or 4.5 participant hours as course credit as well as 4 Euros bonus for their performance in the MMST.

### Outcomes {12}

The primary outcomes are self-reported stress and relaxation measured with a visual analog scale (VAS) at pre- and post-baseline (t2); at the start, middle, and end of the stress induction (t3); at pre- and post-relaxation (t4); at pre- and post-recovery (t5); and in the evening of testing (t6). Secondary outcomes are the physiological parameters, namely heart rate and heart rate variability, tonic skin conductance level as well as the number of non-specific skin conductance responses, systolic and diastolic blood pressure and respiratory rate recorded during the four experimental phases at time points t2 to t5 as well as state mood, and state rumination assessed at four time points (at t2 baseline/pre-stress, at t3 post-stress, at t4 post-relaxation, and at t6 in the evening of testing). Finally, post-event processing will be assessed at t5 after relaxation and at t6 in the evening of testing

### Participant timeline {13}

**Fig. 3:**
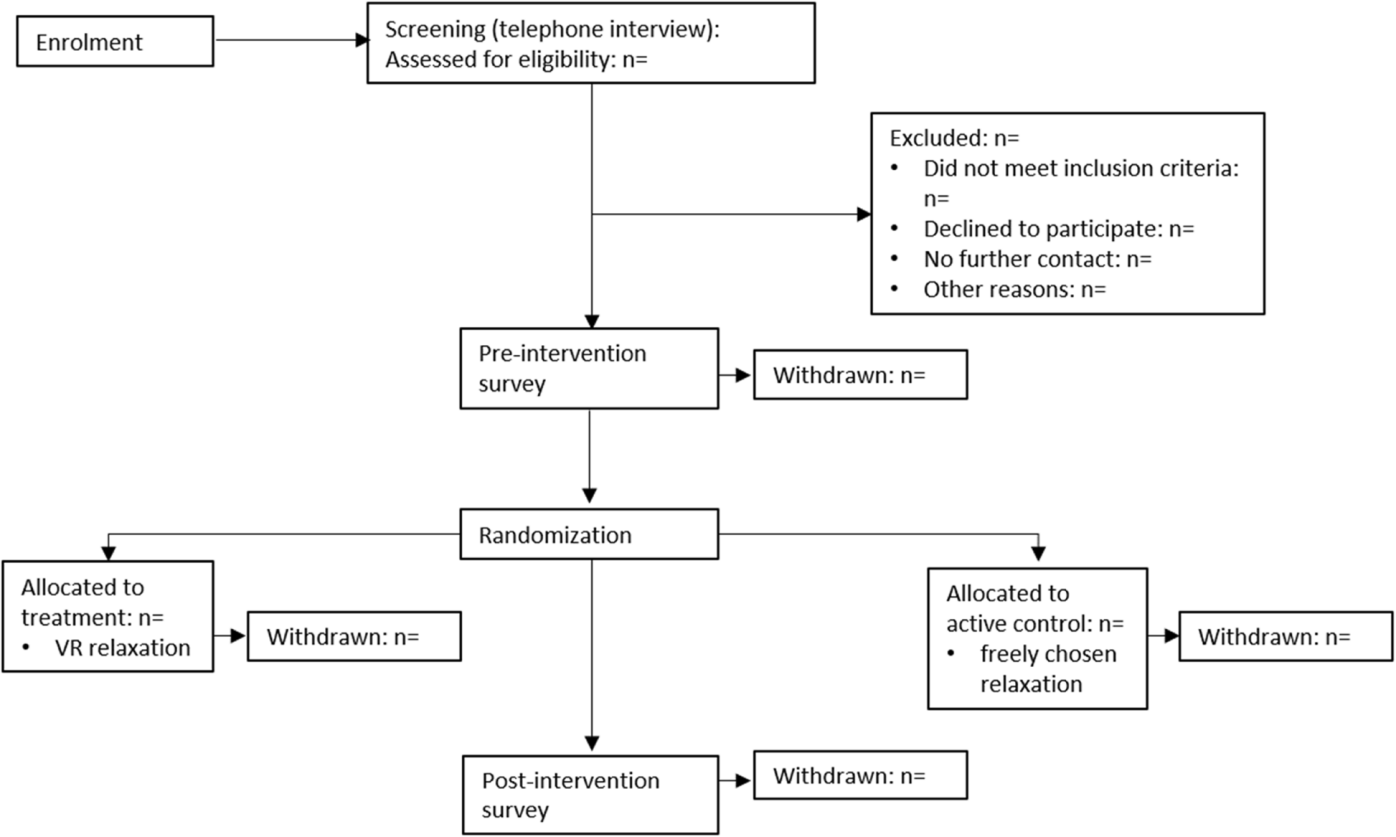
CONSORT flow diagram

Figure 3 gives a CONSORT flow diagram for the schedule from enrollment to the end of participation

A schedule of enrollment, interventions, and assessments is given in Table 1.

**Table 1:** Schedule of enrollment, interventions, and assessments. *VR* virtual reality

	Enrollment	Allocation	Study period					
			Pre	Intervention				Post
**Time point**	***-t***_***1***_	**0**	***t***_***1***_	***t***_***2***_	***t***_***3***_	***t***_***4***_	***t***_***5***_	***t***_***6***_
**Enrollment**								
**Eligibility screen**	X							
**Informed consent**	X							
**Allocation**		X						
**Interventions**								
***Stress induction***					X			
***VR relaxation***						X		
***Freely chosen relaxation***						X		
**Assessments**								
**Self-report measures**								
*Depression, anxiety, and stress*			X					
*Chronic stress*			X					
*Trait rumination*			X					
*Perceived stress*			X					
*Self-efficacy*			X					
*Mindful attention and awareness*			X					
*State stress*				X	X	X	X	X
*State relaxation*				X	X	X	X	X
*State mood*				X	X	X		X
*State rumination*				X	X	X		X
*Post-event processing*						X		X
*Sense of presence in VR*						X		
**Physiological data**								
*Heart rate*				X	X	X	X	
*Heart rate variability*				X	X	X	X	
*Tonic skin conductance level*				X	X	X	X	
*Phasic skin conductance responses*				X	X	X	X	
*Respiration rate*				X	X	X	X	
*Systolic and diastolic blood pressure*				X	X	X	X	

### Sample size {14}

Apriori power analyses were performed for a non-inferiority trial in Sealed Envelope Ltd. 2012 [37]. To achieve a power of 90% at an alpha level of 0.05 assuming a non-inferiority margin of 25% and 80% success in both groups, the estimated required sample size is 88. Considering potential dropouts, we decided to acquire data from 100 participants

### Recruitment {15}

One hundred participants will be recruited from the University of Siegen via internal mailing lists for students. After participants have contacted us via email for study participation, details on the study will be given in a short telephone interview and the aforementioned eligibility criteria will be checked.

### Assignment of interventions: allocation

#### Sequence generation {16a}

Participants will be randomly assigned to the VR group or the active control group according to an externally constructed, randomization plan with a 1:1 ratio. A computerized simple randomization will be implemented based on computer-generated random numbers to control for the equal size of the study groups.

#### Concealment mechanism {16b}

The randomization plan will be password-protected, and the password is only known to the lead investigator and the recruiters. The lead investigator will keep a separate list of the generated randomization plan to guarantee unbiased allocation.

#### Implementation {16c}

Separate individuals will be responsible for randomization, allocation, and data acquisition to avoid potential bias. The lead investigator who will not be involved in participant recruitment and data acquisition will generate the randomization plan. The recruiters of the trial management group will allocate included participants sequentially based on the randomization plan. The experimenters of the trial management group will conduct data acquisition.

### Assignment of interventions: blinding

#### Who will be blinded {17a}

Since participants will be blinded until the onset of the relaxation phase, baseline and stress phases will not be influenced by group assignment. Blinding participants for the relaxation phase will not be possible since either the VR or an own relaxation method will be applied. Similarly, blinding is not possible for the experimenters. The data analysis will comprise two separate processing stages: analysis of raw data in Matlab 2020b (The Mathworks Inc., Natick, MA, USA) and statistical tests in SPSS Statistics 27 (Armonk, NY: IBM Corp.). Missing values and outliers will be treated at the first analysis stage. The data analyst will be blinded until the performance of the statistical test at the second analysis stage.

#### Procedure for unblinding if needed {17b}

Participants will be unblinded immediately before the relaxation phase by revealing their allocation and giving the respective instructions. The data analyst will be unblinded at the second analysis stage.

### Data collection and management

#### Plans for assessment and collection of outcomes {18a}

Physiological data will be recorded using the Biopac MP160 recording system (BIOPAC Systems Inc., Goleta, CA, USA) with a sampling rate of 2000 Hz, using the ECG 100C, the GSR 100C, and the RSP 100C amplifiers, and the wirerless Bionomadix transmitters. Three pre-gelled Ag/Ag-Cl ECG electrodes will be placed in lead II ECG configuration. For skin conductance, two self-adhesive Ag/AgACl electrodes prefilled with an isotonic electrolyte medium will be attached to the thenar and hypo-thenar of the non-dominant hand. The BioNomadix respiration transducer will be fastened around either the abdomen or chest depending on the participant’s preferred respiration type. Blood pressure will be acquired with the Dinamap Pro 300 (General Electric Deutschland Holding GmbH, Frankfurt am Main, Germany). A blood pressure cuff will be fastened around the participant’s non-dominant arm. Blood pressure measurement will be automatically triggered via a serial port using a Presentation script.

#### Plans to promote participant retention and complete follow-up {18b}

The duration of the intervention is very short taking approximately 3 h. To increase the likelihood of participation at time point t6 in the evening of testing, a reminder will be sent around via email after the laboratory testing session. Participants may withdraw from the study at any time. The respective data will only be considered if participants finished laboratory testing; otherwise, participants will be excluded from all analyses.

#### Data management {19}

All physiological data recorded with the Biopac MP160 recording system as well as ratings and questionnaires assessed with the software Presentation and the online survey tool limesurvey are electronically logged. The only information that will be acquired in paper form and that will be entered manually is the protocol sheet with the blood pressure measurements and two additional questionnaires. One research assistant from the recruiters will enter them, another one will control for errors in data entry. Participants’ written consent forms will be stored for 10 years in numerical order in a locked cabinet.

#### Confidentiality {27}

Collected data will be identified by a coded ID number to maintain participant confidentiality. All records that contain names or other personal identifiers, such as informed consent forms, will be stored separately from study records identified by code number

#### Plans for collection, laboratory evaluation, and storage of biological specimens for genetic or molecular analysis in this trial/future use {33}

No biological specimens will be collected.

### Statistical methods

#### Statistical methods for primary and secondary outcomes {20a}

Analysis of acquired raw data will be conducted in Matlab 2020b (the Mathworks Inc., Natick, MA, USA). All physiological data will be visually screened. ECG data will be analyzed with the Pan-Tompkins algorithm for QRS detection [38, 39]. Heart rate will be estimated as the number of beats per minute. Heart rate variability will be estimated using root mean square of successive differences (RMSSD, [40]). The tonic skin conductance level and the number of non-specific phasic skin conductance responses will be estimated using the matlab toolbox Ledalab [41]. Respiratory rate will be estimated per minute using a custom-made matlab script that band pass filters the data to frequencies of 0.05 to 1 Hz before detection of inhalation peaks and exhalation troughs. SPSS Statistics 27 for Windows (Armonk, NY: IBM Corp.) will be used for statistical analyses. To test the effects of time (within-subject factor), group (between-subject factor), and the time by group interaction, repeated measures ANOVAs will be performed. Additional two sample *t* tests will evaluate the group differences at relaxation and post-relaxation.

#### Interim analyses {21b}

Study data will be analyzed by the lead investigator in the middle (interim analysis at *N* = 50) and after completion of data acquisition. Planned analyses are described in SPIRIT item 20a. There will be no formal stopping rules since we do not anticipate problems detrimental to participants. Data collection will be stopped when at least fifty participants will be included in each group (VR, active control). The lead investigator and the principal investigator will make the final decision to terminate the data collection.

#### Methods for additional analyses (e.g., subgroup analyses) {20b}

To explore if the current stress level, trait mindfulness, and trait self-efficacy impact the success of the applied relaxation method, we will include them as covariates in the above models. No additional subgroup or adjusted analyses are planned.

#### Methods in analysis to handle protocol non-adherence and any statistical methods to handle missing data {20c}

Missing values will be replaced by multiple imputation; missing segments in the physiological data will be discarded.

#### Plans to give access to the full protocol, participant-level data, and statistical code {31c}

Version 1.0 of the study protocol is available from the preprint server PsyArXiv [42]. The datasets generated and analyzed during the current study as well associated statistical codes will be available after the publication of the study results upon request from the corresponding author (contact miriam.kampa@uni-siegen.de).

### Oversight and monitoring

#### Composition of the coordinating center and trial steering committee {5d}

The steering committee consisting of all lead and principal investigators of the two universities as well as the industrial cooperation partners will have monthly, virtual meetings to address goals and progress of the overall multidisciplinary project “NOSTRESS.” Public involvement will be realized in the form of public, interactive virtual meetings with external experts to discuss issues of stress, health, and technology organized by one of the cooperation partners. The lead investigator of the present RCT trial (subproject of “NOSTRESS”) was responsible for the study design, prepared the protocol, and will organize bi-weekly virtual meetings with the trial management group. In this bi-weekly meetings, the trial management group will report on problems and progress of ongoing data collection. The trial management group will consist of seven research assistants: three of them will be responsible for participant recruitment and screening (referred to as recruiters), and four will conduct the laboratory testing sessions (referred to as experimenters). A technical support team consisting of two experienced post-doctoral researchers will assist in the occurrence of technical problems and participate in the bi-weekly meetings, whenever necessary. The lead investigator will prepare the randomization plan and will monitor the acquired data for completeness and validity (e.g., corrupt recordings). The lead investigator together with the principal investigator will decide on trial termination and will write up study results for publication.

#### Composition of the data monitoring committee, its role, and reporting structure {21a}

There will be no independent data monitoring committee since this will be a low-risk intervention.

#### Adverse event reporting and harms {22}

We do not expect any serious adverse events or adverse events from the relaxation interventions. The stress induction paradigm has been applied to more than two hundred healthy participants of the same age range at different study sites without the occurrence of any serious adverse events [30, 42, 43]. The only minor adverse event that we observed in former studies is the experienced psychological distress causing approximately 3% of study participants to withdraw from the study. The trial management group will document any adverse events and report it to the lead investigator. Unexpected, serious, and severe events occurring during the trial will be reported to the local ethics committee that approved the study. An intern physician can always be contacted concerning health-related questions.

#### Frequency and plans for auditing trial conduct {23}

An audit may be performed by the members of the steering committee not involved in the conduct of the study to evaluate compliance with the study protocol.

#### Plans for communicating important protocol amendments to relevant parties (e.g., trial participants, ethical committees) {25}

Modifications to the study protocol will be communicated and discussed with the steering committee. In the case of larger modifications to the experimental design, an amendment of the study protocol will be sent to the local ethics committee. Any deviations from the study protocol will be fully documented using a breach report form. The protocol will be updated in the clinical trial registry (ISRCTN).

#### Dissemination plans {31a}

The lead investigator will communicate the results of the trial to the steering committee. The trial results will be published in a peer-reviewed journal. Data and materials are available upon request from the corresponding author

## Discussion

A multimodal VR environment for relaxation and stress reduction was developed and will be tested against a freely chosen relaxation method in a non-inferiority trial. In order to test the efficacy of the relaxation VR in a stressful situation, participants are acutely stressed before the relaxation phase. The major advantage of the current relaxation VR is that it does not require prior knowledge or training and can directly be applied for stress reduction and relaxation. Compared to common practices like meditation or mindfulness-based attention, the VR is time-saving and the initial hurdle to learn relaxation practices is low.

Since contemporary VRs are becoming increasingly more realistic and immersive, this allows the use in psychotherapeutic interventions. As an example, VRs have been explored as an alternative to in vivo exposure therapy in a range of specific phobias yielding higher compliance rates [44]. Applied as a tool for relaxation, VRs can shield participants from distraction and assist the practice of mindfulness [12]. The scope of a VR environment for relaxation is not only to influence ongoing processes of relaxation but ideally to train sustainable relaxation skills in the users. With the technique of the visual and haptic intervention on respiration, relaxation processes can be influenced online and learning can be facilitated.

One approach to support transfer to real life could be a stepwise elimination of components of the VR until relaxation is solely performed without the use of the VR. Since the complete experimental setup of the current study is quite laborious, rather immobile and thus not suitable for a broad distribution, one of our cooperation partners will test three different, reduced versions of the presented relaxation system in a longitudinal, qualitative study. Learning relaxation with the system will be monitored at three learning stages comprising regular practice with the VR (stage 1), with the VR and vibration pillow (stage 2), and ultimately with only the pillow as a reminder of the intervention (stage 3). Another approach to improve transfer to real life, but also adherence to the VR, would be the implementation of a mobile app version of the VR environment. Of course, a mobile app version would be less realistic, but for a trained user, it might be a helpful cue and would increase accessibility. Studies on existing mobile apps for mindfulness training have achieved benefits in mood and increases in mindfulness [45–47]. In summary, if evaluated successfully, the virtual reality developed as part of our current study might be a useful tool to facilitate relaxation and stress reduction without prior training in stressful situations*.*

## Trial status

This trial was registered with the ISRCTN Registry on January 22, 2021 (Trial ID: ISRCTN11162338, protocol version number 1.0, https://www.isrctn.com/ISRCTN11162338). Recruitment of participants started in October 2020 and ended in May 2021.

## Abbreviations

DASS: Depression, Anxiety, Stress Scales
ECG: Electrocardiogram
GSR: Galvanic skin conductance response
Igroup: Questionnaire on experienced presence in the VR
MAAS: Mindful Attention and Awareness Scale
MDBF: Mood State Questionnaire
MMST: Mannheim Multicomponent Stress Test
PASAT-C: Paced Auditory Serial Addition Task
PEPR: Post-event processing
PSS-10: Perceived Stress Scale
VAS: Visual analog scale
VR: Virtual reality
RCT: Randomized controlled trial
RMSSD: Root mean square of successive differences (in beat-to-beat intervals)
RSQ: Response Styles Questionnaire
s: State
SWE: Self-Efficacy Questionnaire
TICS: Trier Inventory for Chronic Stress
